# Sputum Glutaredoxin 1 and Protein S-Glutathionylation in COPD

**DOI:** 10.3390/antiox15030330

**Published:** 2026-03-06

**Authors:** Ine Kuipers, Renske Krijgsman, Renaud Louis, Jean-Louis Corhay, Thibault Azevedo Mendes, Guy G. Brusselle, Ken R. Bracke, Niki L. Reynaert

**Affiliations:** 1 Department of Respiratory Medicine, Nutrition and Translational Research in Metabolism (NUTRIM), Maastricht University Medical Center+, 6229 ER Maastricht, The Netherlands; ine_kuipers@hotmail.com (I.K.); renske.krijgsman1@gmail.com (R.K.); 2 CHU Sart-Tilman, I GIGA Research Group, Department of Pulmonary Medicine, University of Liège, 4000 Liège, Belgium; r.louis@chu.ulg.ac.be (R.L.); jlcorhay@chu.ulg.ac.be (J.-L.C.); tmmendes@student.uliege.be (T.A.M.); 3 Department of Respiratory Medicine, Ghent University Hospital, 9000 Ghent, Belgium; guy.brusselle@uzgent.be (G.G.B.); ken.bracke@ugent.be (K.R.B.)

**Keywords:** glutaredoxin, COPD, exacerbation, sputum, extracellular, secretion

## Abstract

Alterations in glutathione and its metabolism contribute to oxidative stress in COPD, but the role of S-glutathionylation (PSSG) and its major regulator glutaredoxin 1 (Grx1) remains unclear. This study investigated the Grx1/PSSG axis in sputum of COPD patients and its associations with lung function and inflammation, as well as Grx1 secretion in mouse models and in cell culture. In patients with an acute exacerbation, PSSG levels were significantly decreased in sputum, while Grx1 protein and total Grx activity were increased compared to stable COPD. No differences were observed between healthy smokers and stable patients. PSSG levels correlated negatively with sputum neutrophils, IL-8 and IL-1β, but positively with lung function parameters, whereas Grx1 showed the opposite pattern. Enhanced Grx1 levels were also detected in bronchoalveolar lavage fluid from mice exposed to cigarette smoke or chronic pulmonary inflammation. Moreover, epithelial cells and macrophages secreted Grx1 in response to pro-inflammatory mediators, and Grx1 modulated expression of MMPs by macrophages in vitro and in vivo. In conclusion, this study identifies the Grx1/PSSG redox axis as a potential important factor in COPD pathogenesis, especially during exacerbations. Further research should examine in more detail the intricate relation of extracellular Grx1 with lung function and inflammation.

## 1. Introduction

Chronic Obstructive Pulmonary Disease (COPD) is the third leading cause of death. It is hallmarked by small airway abnormalities and/or emphysema, which are associated with irreversible airflow limitation and symptoms such as cough and dyspnea [1,2]. Periodic disease flare-ups, or exacerbations, significantly contribute to disease progression and reduced quality of life. Smoking is the most important risk factor for the development of COPD. Notably, even after smoking cessation, inflammation persists, and lung function continues to decline. Cigarette smoke contains 10^16^ free radicals per cigarette [3] and promotes the activation and recruitment of macrophages, neutrophils and adaptive immune cells into the lungs. This leads to chronic inflammation, which drives tissue remodeling. The inflammatory cells, mitochondrial dysfunction and activation of NADPH oxidases represent important endogenous sources of oxidants in COPD patients. Together, these processes overwhelm the pulmonary antioxidant defense system and contribute to oxidative stress and tissue damage [4].

The tripeptide glutathione (GSH) is the most important component of the pulmonary antioxidant defense system. Total glutathione levels in the epithelial lining fluid are approximately 140-fold higher than in plasma [5]. Acute exposure to cigarette smoke depletes GSH levels. However, during chronic smoking, GSH concentrations increase as an adaptive response to oxidative stress. This increase is mediated by upregulation of the rate-limiting enzyme in GSH synthesis, γ-glutamylcysteine ligase, through activation of the transcription factor Nrf-2 [6]. In COPD, the activation of this adaptive antioxidant mechanism is impaired and fails to provide adequate protection against oxidant-induced damage.

GSH, together with its redox cycle partners, maintains the reduced state of protein thiol groups. This occurs either through direct scavenging of oxidants or through the reversible covalent binding of GSH to these protein thiols [7]. The latter formation of mixed disulfides between protein thiols and GSH occurs under physiological conditions and is induced upon mild oxidative stress. This reversible post-translational modification is known as S-glutathionylation or S-glutathiolation (PSSG) [8,9]. PSSG not only protects the targeted protein thiol groups from further irreversible oxidations but can also modulate protein function, thereby influencing cellular signaling pathways [10]. For example, inflammatory signalling pathways such as NF-κB and AP-1 are inhibited through PSSG of, amongst others, Inhibitory kappa B kinase β (IKKβ), and p50 and c-jun subunits of the transcription factors [11,12,13].

Glutaredoxins (Grx), or thioltransferases, are redox enzymes that reverse PSSG under physiological conditions and therefore play a key role in redox regulation [8]. Several mammalian Grx isoforms have been identified, of which Grx1 is the best characterized. Grx1 is localized in the cytosol and the mitochondrial intermembrane space, and is responsible for most deglutathionylation activity in mammalian cells [8].

Despite extensive research on GSH/GSSG imbalances in COPD, and therapeutic efforts to augment GSH levels in COPD and other chronic lung diseases, the Grx1-PSSG axis remains only sparsely investigated. Peltoniemi et al. reported decreased Grx1 levels in the lungs of COPD patients, along with a reduced number of Grx1-positive macrophages that positively correlated with lung function [14]. In line, we and others reported reduced Grx1 levels in lungs of mice exposed to cigarette smoke [15,16]. The direction in which Grx1 modulated smoke-induced inflammation appears to be dependent on the duration of the exposure, with *Glrx1*-deficient mice demonstrating aggravated neutrophilia after 3 days of exposure, whereas they were shown to be protected from neutrophilia after exposure for four weeks [15,16].

Intriguingly, although Grx1 lacks a signal peptide for secretion, it has been detected extracellularly in plasma [17] and sputum. In sputum of patients with asthma, we previously reported increased levels of Grx1, especially in patients with eosinophilic and paucigranulocytic inflammation, which negatively correlated with lung function [18]. In COPD, increased sputum Grx1 levels were observed during disease exacerbations compared with non-smoking controls [14]. Surprisingly, sputum Grx1 levels were not significantly different between exacerbation and stable disease. Furthermore, Grx1 sputum levels were not examined in relation to Grx enzymatic activity, PSSG levels, or inflammatory cells or mediators.

Given these previous reports and outstanding questions on the significance of sputum Grx1 in COPD, the aim of the current study was to investigate the Grx1-PSSG axis in induced sputum of COPD patients compared to controls and to investigate the correlation thereof with measures of lung function and inflammation. Secondly, the secretion of Grx1 was examined in mouse models of COPD, and its relevance was tested in cell culture.

## 2. Materials and Methods

### 2.1. Study Design and Subject Characteristics

Induced sputum samples were collected from 16 patients with stable COPD, 14 patients with COPD experiencing an acute exacerbation (on day 1 or 2), and 25 healthy control subjects. All COPD patients met the diagnostic criteria established by the Global Initiative for Chronic Obstructive Lung Disease (GOLD) guidelines [1,19]. Stable patients had not experienced an upper respiratory tract infection or an exacerbation requiring changes in maintenance therapy, oral corticosteroids, or antibiotic treatment for at least 12 weeks prior to the study. Among the patients with exacerbations, four were recruited during outpatient consultations, and 9 were recruited upon hospital admission to the service of pneumology of CHU of Liège, Belgium. An acute exacerbation of COPD was defined as an event in the natural course of the disease characterized by a change in the patient’s baseline dyspnoea, cough and/or sputum that was beyond normal day-to-day variations, acute in onset, and warranted a change in regular medication [1]. Exacerbations were treated according to good clinical practice. The control subjects had no history of asthma or chronic bronchitis and had not experienced any bronchial or respiratory tract infection during the 8 weeks preceding the study. Their pulmonary function was within the normal range. This study was approved by the local ethics committee at Liège University, Belgium (2015/193).

### 2.2. Sputum Induction and Processing

After premedication with 400 μg inhaled salbutamol administered by metered dose inhaler (MDI), sputum induction was performed by inhalation of hypertonic saline (5% NaCl) in subjects with a post-salbutamol FEV_1_ ≥ 65% predicted and isotonic saline (0.9% NaCl) in those with FEV_1_ < 65% predicted. Saline was administered in combination with additional salbutamol using an ultrasonic nebulizer (Ultra-Neb 2000; Devilbiss, Somerset, PA, USA) set at an output of 0.9 mL/minute, as previously described [20]. Subjects inhaled the aerosol for three consecutive periods of 5 min (total duration 15 min). For safety reasons, FEV_1_ was monitored every 5 min, and the induction was stopped if FEV_1_ decreased by more than 20% from post-bronchodilator values. The whole sputum was collected in a plastic container, weighed, and homogenized by adding three volumes of phosphate-buffered saline, vortexed for 30 s, and centrifuged at 800× *g* for 10 min at 4 °C. The supernatant was separated from the cell pellet, which was suspended in Roswell Park Memorial Institute (RPMI) 1640 medium supplemented with 100 U/mL penicillin, 100 μg/mL streptomycin, and centrifuged at 400× *g* for 10 min at 4 °C. Cells were washed once more with RPMI 1640 containing antibiotics. Squamous and total cell counts, and cell viability (trypan blue exclusion) were determined using a manual hemocytometer. Differential cell counts were performed on cytospin preparations stained with Diff-Quick, with 400 cells counted per sample.

### 2.3. Murine Studies

Eight-week-old male C57BL/6 mice (*n* = 20) were obtained from The Jackson Laboratory (Bar Harbor, ME, USA). Additionally, male *Glrx1*^−/−^ mice (*n* = 10) were used (gifted by Dr. Ho, Wayne State University, Detroit, MI, USA) [16]. The mice received food and water ad libitum and were exposed whole body to the tobacco smoke of 5 reference cigarettes 3R4F without filter (University of Kentucky, Lexington, KY, USA) four times a day with 30 min smoke-free intervals, 5 days a week, for 4 weeks. During the exposure an optimal smoke-to-air ratio of 1:6 was obtained as previously described [21]. The control group was exposed to room air. The local ethics committee for animal experimentation of the faculty of Medicine and Health Sciences of Ghent University, Belgium, approved the in vivo manipulations (ECD09/20).

SPC-TNFα mice overexpress the human *TNFα* gene in surfactant protein C (SPC) producing cells (alveolar type II cells) and display chronic pulmonary inflammation, emphysema and lung fibrosis [22]. Mice received water and food ad libitum. For experiments, 12-month-old male and female SPC-TNFα (*n* = 11) and WT littermate control mice (*n* = 8) were used. The animal protocol was approved by the Animal Care and Use Committee of the National Institute on Aging (Baltimore, MD, USA) (352-TGB-2018).

After euthanasia with an overdose of pentobarbital, a cannula was inserted into the trachea. Bronchoalveolar lavage (BAL) was performed as previously described [16]. The left lung was inflated and fixated by intratracheal infusion of 4% paraformaldehyde (PFA) under a constant pressure of 20 cmH_2_O above the highest point of the lung and embedded in paraffin.

### 2.4. Grx1 Immunohistochemistry and Immunofluorescence

Four µm paraffin sections were deparaffinized and rehydrated, and antigen retrieval was performed by boiling the sections in 1 mM EDTA (pH 8.0) for 10 min. After blocking with 5% bovine serum albumin (BSA) in Tris-buffered saline (TBS) for 20 min, slides were incubated with an antibody against Grx1 (R&D Systems) for 1 h. Sections were subsequently incubated with a biotin-labelled secondary antibody (DAKO) for 30 min. Staining was visualized using the Vectastain ABC/AP system and Vector Blue as substrate. Nuclei were counterstained with Nuclear Fast Red, after which sections were dehydrated and mounted with coverslips.

BAL fluid samples were centrifuged onto microscope slides, and cytospins were fixed with 4% PFA for 10 min at RT. After permeabilization and blocking non-specific binding sites with 0.1% Triton X-100 and 1% BSA in phosphate-buffered saline (PBS) for 20 min, slides were incubated with a primary antibody against Grx1 (IMCO, Stockholm, Sweden) for 1 h, followed by an Alexa Fluor 555-labelled anti-goat secondary antibody for 1 h. Nuclei were counterstained with DAPI, and slides were mounted with coverslips. Semi-quantitative analysis of staining intensity was performed by using Image J 1.8 Software. Mean red fluorescence intensity (Grx1 staining) was normalized to mean blue fluorescence intensity (nuclear staining).

### 2.5. Cell Culture

The murine macrophage cell line J774.1 was obtained from the American Type Culture Collection (ATCC) (Manassas, VA, USA). Cells were maintained in Dulbecco’s Modified Eagle’s Medium (DMEM) GlutaMax high-glucose medium supplemented with 10% fetal bovine serum (FBS, Bodinco, Alkmaar, The Netherlands), 100 U/mL penicillin and 100 µg/mL streptomycin (pen/strep). H292 human bronchial epithelial cells were purchased from the ATCC and cultured in RPMI 1640 supplemented with 10% FBS, 2 mM L-glutamine and pen/strep. Prior to stimulation experiments, cells were serum-starved in phenol red-free RPMI 1640 medium supplemented with 0.5% FBS, 2 mM L-glutamine and pen/strep.

### 2.6. Cell Stimulations

Filters were removed from 3R4F research cigarettes (University of Kentucky, Lexington, KT, USA), and cigarette smoke extract (CSE) was prepared according to Carp et al. [23]. Briefly, a linear pump was used to bubble air (2 mL/s) through 2 mL HBSS for 5 s, followed by a 5 s pause. CSE was protected from light, sterile-filtered and defined as 100%. Cells were stimulated within 15 min with indicated concentrations of CSE.

Cells were furthermore treated with lipopolysaccharide (LPS; E. coli O26:B6, Sigma, Burlington, MA, USA), phorbol 12-myristate 13-acetate (PMA, Sigma), recombinant human TNFα (R&D Systems, Minneapolis, MN, USA), or recombinant human Grx1 (Abfrontier, Baileys Harbor, WI, USA).

### 2.7. Western Blotting for Grx1 and PSSG

Lung tissue and cultured cells were lysed in buffer containing 137 mM Tris-HCl (pH 8.0), 130 mM NaCl, 1% NP-40 and protease inhibitor cocktail (Roche, Mannheim, Germany). After determining the protein content, Laemmli sample buffer containing DTT was added to 25 μg of lung or cell culture homogenate and boiled for 10 min. For sputum samples, 25 μL was mixed directly with DTT containing Laemmli buffer. For detection of PSSG, lung tissue lysates were prepared in non-reducing Laemmli sample buffer. Samples were loaded onto a 16.5% polyacrylamide gel and transferred onto a nitrocellulose membrane. Membranes were blocked at RT for 1 h in 5% BSA in Tris-buffered Saline (TBS) containing 0.05% Tween 20 (TBST). After two washes in TBST, membranes were incubated overnight at 4 °C with primary antibody against Grx1 (IMCO), GSH (Virogen, Newport, RI, USA), GAPDH (Cell Signalling Technologies, Danvers, MA, USA) or α-tubulin (Cell Signalling Technologies). After three washes with TBST, membranes were incubated with peroxidase-conjugated secondary antibodies for 1 h at RT. After three washes with TBST, conjugated peroxidase was detected by chemiluminescence using the Pierce ECL Western Blotting Substrate (Thermo Scientific, Rockford, IL, USA). Quantification was performed using a Biorad scanner. For induced sputum samples, results were expressed as arbitrary units relative to human recombinant Grx1 (Abfrontier) included on each gel. For lung tissue and cell lysates, protein levels were normalized to GAPDH or a-tubulin and expressed as arbitrary units.

### 2.8. Grx Activity Assay

A total of 100 μL of sputum was incubated with an equal volume of reaction buffer containing 137 mM Tris-HCl, pH 8.0, 0.5 mM glutathione, 1.2 U glutathione disulfide reductase (Roche), 0.35 mM NADPH, 1.5 mM EDTA (pH 8.0), and 2.5 mM cysteine-SO_3_. Consumption of NADPH was followed spectrophotometrically at 340 nm over 10 min. The specific enzymatic activity was calculated by subtracting the reaction rate measured in the absence of the substrate (cysteine-SO_3_) from the rate obtained in its presence. Enzyme activity is expressed as µmol NADPH consumed per minute [24].

### 2.9. Quantitative Determination of S-Glutathionylated Proteins Using 5,5′-Dithio-bis(2-nitrobenzoic Acid) (dTNB)

A total of 200 μL of sputum was precipitated with acetone for 20 min at −20 °C and centrifuged for 5 min at 3000× *g*. Pellets were resuspended and sonicated in 200 μL of ice-cold extraction buffer containing 0.2% Triton-X 100 and 0.6% sulfosalicyclic acid in 0.1 M potassium phosphate buffer with 5 mM EDTA disodium salt (KPE), pH 7.5. After two freeze–thaw cycles, samples were centrifuged at 3000× *g* for 4 min at 4 °C. To release glutathione (GSH) from proteins, the pellet was treated with 100 μL of 1% NaBH_4_ in water and subsequently neutralized with 40 μL of 30% metaphosphoric acid. Samples were centrifuged at 1000× *g* for 15 min, and the supernatant was collected to determine the GSH content using the DTNB–GSSG reductase recycling method [25]. For the assay, 20 μL of KPE buffer, GSH standards or samples were pipetted into a 96-well microtiter plate and freshly prepared, equal volumes of DTNB and GSSG reductase were added in the dark. After 30 s, β-NADPH was added to initiate the conversion of DTNB to TNB. Absorbance at 412 nm was measured every 30 s for 2 min. A standard curve was generated using a range of GSH concentrations. For each sample, NaBH_4_ treatment was omitted in parallel as a negative control. GSH levels were normalized to total protein content and data expressed as nmol GSH per milligram of protein.

### 2.10. Grx1 ELISA

Grx1 levels were measured in BAL fluid or cell culture supernatant using the anti-human Grx1 capture antibody and the human biotinylated anti-Grx1 detection antibody (IMCO) according to the manufacturer’s instructions.

### 2.11. Luminex Analyses

A Bio-Plex human cytokine 27-plex panel was used to quantify concentrations of 27 cytokines, chemokines and growth factors in sputum supernatants. Assays were formed as described by the manufacturer’s instructions. The analysis was performed with a Luminex 100 IS 2.3 system using the Bio-Plex Manager 4.1.1. software.

### 2.12. QPCR

Total RNA was extracted from lung tissue using the RNeasy Mini kit (Qiagen, Valencia, CA, USA) and from cells in culture using the HiPure RNA isolation kit (Roche). Equal amounts of RNA were reverse-transcribed into cDNA using the Transcriptor cDNA synthesis kit (Roche). Details on primers used can be found in the supplemental table. Quantitative PCR was performed on an ABI 7900HT apparatus (Applied Biosystems, Waltham, MA, USA) using SYBR green dye (Applied Biosystems). Gene expression levels were normalized to the housekeeping genes *RPL13A* or *HPRT*, as indicated. Relative mRNA expression was calculated using the DDCT method.

### 2.13. Statistical Analyses

SPSS (version 15) was used for data analyses. Unless indicated otherwise, results are presented as mean and standard deviation. For human samples, between-group comparisons were analyzed using the Kruskal–Wallis test, followed by pairwise comparisons with the Mann–Whitney U-test. Categorical variables were compared using the chi-square test, and correlations between continuous variables were assessed using Spearman’s rho. Non-parametric tests were applied due to the relatively small sample size. In vivo and in vitro experiments were analyzed using Student’s *t*-test or one-way ANOVA with Bonferroni post-hoc correction, as appropriate. A *p*-value < 0.05 was considered statistically significant.

## 3. Results

### 3.1. Biochemical Assessment of the Grx1–PSSG Axis in Induced Sputum Samples of COPD Patients

Both control and COPD groups were well matched for age, sex and BMI. Only patients experiencing an exacerbation were significantly older than non-smoking controls (Table 1). Patients had smoked more pack years compared to smoking controls, but the proportion of ex-smokers was similar in both groups (COPD: 81% ex-smokers; smoking controls: 75% ex-smokers). As expected, COPD patients showed impaired lung function compared to both healthy non-smokers and healthy smokers as reflected by lower FEV_1_% predicted, lower FVC % predicted and a reduced FEV_1_/FVC ratio. Analysis of induced sputum demonstrated that stable COPD patients had a significantly higher percentage of neutrophils compared to smoking controls. Moreover, sputum from COPD patients experiencing an exacerbation contained higher levels of IL-1β compared to sputum from patients with stable disease (Table 1).

**Figure 1:**
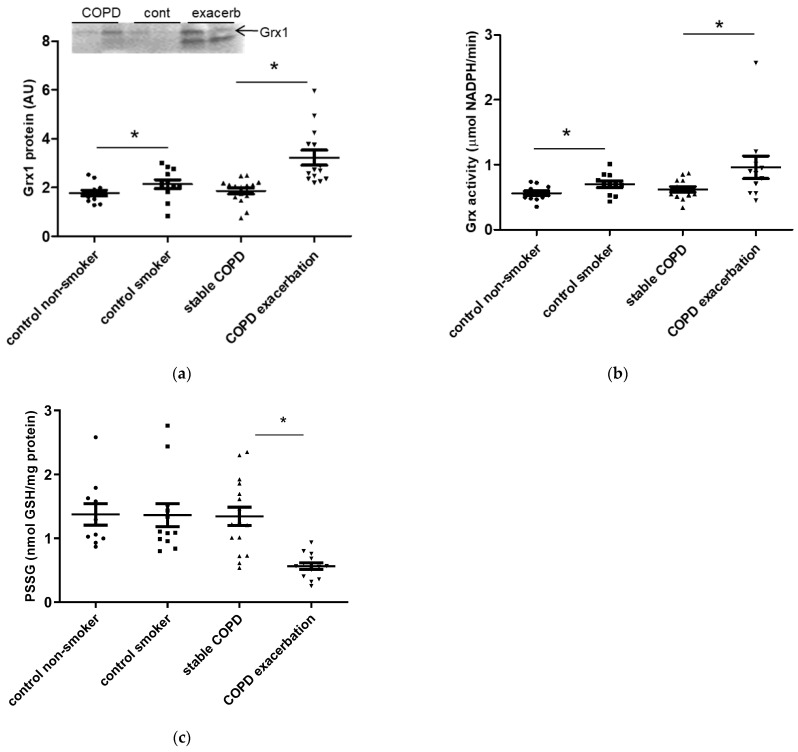
The Grx1–PSSG axis in induced sputum of control non-smokers, control smokers, stable COPD patients and COPD exacerbators. (**a**) Grx1 protein level in induced sputum assessed by Western blot. Data are expressed in arbitrary units (AU) where Grx1 levels were corrected for a sample of recombinant human Grx1 on each gel and represented as individual data points, mean ± SD. The insert is a representative Western blot where the arrow indicates the MW of intact Grx1. Note that a smaller size Grx1 can be detected in sputum of patients experiencing an exacerbation. (**b**) Total Grx activity in induced sputum expressed as mmol NADPH per minute and represented as individual data points, mean ± SD. (**c**) Level of protein S-glutathionylation (PSSG) in induced sputum expressed as nmol GSH released per mg protein and represented as individual data points, mean ± SD. * represents *p* < 0.05.

In induced sputum, the level of Grx1 protein detected by Western blot was increased in control smokers compared to control non-smokers, and in COPD patients with an exacerbation compared to stable disease (Figure 1a). Grx1 protein levels did not differ between control smokers and stable COPD patients. Interestingly, in sputum of all COPD patients with an exacerbation, an additional lower molecular weight band was observed (Figure 1a, insert). Measurements of Grx activity paralleled the changes observed in Grx1 protein levels in sputum. Grx activity was increased in sputum of control smokers compared to non-smokers, and also in COPD patients during an exacerbation compared to stable disease (Figure 1b). No significant difference in sputum Grx activity was detected between healthy smokers and stable COPD patients. PSSG levels in induced sputum were significantly decreased in COPD patients experiencing an acute exacerbation compared to those in patients with stable disease (Figure 1c). Importantly, in agreement with the function of Grx to remove GSH from proteins, significant negative correlations were observed between PSSG and Grx1 protein levels (R= −0.667, *p* < 0.001), as well as between PSSG levels and total Grx activity (R= −0.300, *p* = 0.042) across the whole study population (Supplemental Figure S1).

### 3.2. Correlations of the Grx1–PSSG Axis in Induced Sputum

In addition to the increased protein levels of Grx1 and Grx activity observed in sputum of smokers, both parameters showed significant positive correlations with pack years smoked across the entire study population (Grx1 protein levels: R = 0.311, *p* = 0.030; Grx activity: R = 0.355, *p* = 0.015). On the other hand, PSSG levels were negatively correlated with pack years smoked (R = −0.357, *p* = 0.013).

When assessing the relations between the Grx1–PSSG axis and lung function parameters, PSSG levels were positively correlated with FEV_1_% predicted (R = 0.372, *p* = 0.007), FVC% predicted (R = 0.372, *p* = 0.008), as well as FEV_1_/FVC (R = 0.336, *p* = 0.016) in the overall study population. Grx1 protein levels, on the other hand, were negatively correlated with FVC % predicted (R = −0.336; *p* = 0.015) and FEV_1_/FVC (R = −0.436; *p* = 0.001). No significant correlations were found between Grx activity and lung function parameters in the whole study group. Moreover, when COPD patients were considered separately, no significant associations were found between Grx1, Grx activity, or PSSG levels and lung function indices.

Given the known role of Grx and PSSG in modulation of inflammatory pathways, their associations with sputum cell profiles and levels of inflammatory mediators were next assessed. These analyses revealed a significant positive correlation between sputum PSSG levels and the percentage of macrophages (R = 0.535; *p* < 0.001) and a negative correlation with the percentage of neutrophils (R = −0.534; *p* < 0.001) in the whole study group. These correlations remained significant when the analysis was restricted to stable patients (R = 0.733, *p* = 0.001; R = 0.699, *p* = 0.003; R = −0.709, *p* = 0.002, respectively). PSSG levels furthermore negatively correlated with sputum concentrations of IL-1β and IL-8 in the whole study group (R = −0.484, *p* = 0.003; R = −0.475, *p* = 0.003, respectively). While neither Grx1 levels nor Grx activity correlated significantly with cells in induced sputum, Grx1 protein levels were positively correlated with the pro-inflammatory cytokines IL-1β (R = 0.486, *p* = 0.003) and IL-8 (R = 0.427, *p* = 0.009) in the whole study group.

### 3.3. Grx1 in BALF in Mouse Models of COPD

**Figure 2:**
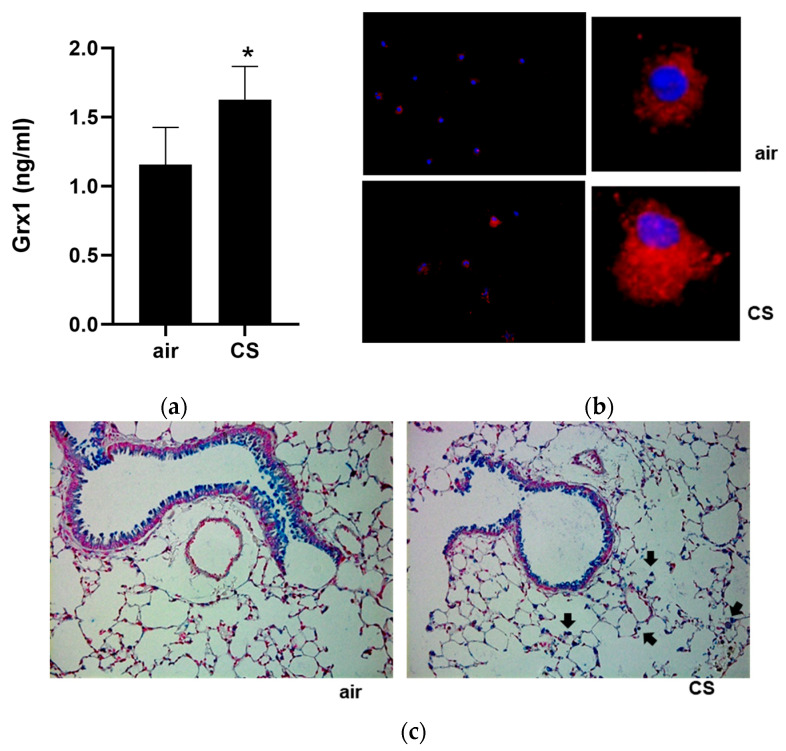
Increased expression and release of Grx1 into the BALF of smoke-exposed mice. (**a**) BALF Grx1 levels in air- (*n* = 5) and CS-exposed (*n* = 5) mice. (**b**) Representative image of Grx1 IF on BAL cells of air- and CS-exposed mice (red: Grx1; blue: nuclei). (**c**) Representative image taken at 20× of Grx1 immunohistochemistry on lung tissue of an air- and smoke-exposed mouse (Grx1: blue; nuclei: red). Arrows indicate alveolar macrophages. Data are expressed as mean ± SD, and * denotes *p* < 0.05 vs. representative control.

It was next examined whether chronic cigarette smoke exposure in mice would reproduce the smoking-associated changes in sputum Grx1 observed in humans. Consistent with the human data, BALF from mice chronically exposed to cigarette smoke for four weeks contained significantly more Grx1 protein compared to air-exposed controls (Figure 2a). In contrast to previous reports describing reduced pulmonary Grx1 expression and total Grx activity following chronic smoke exposure in mice [15,16], lavaged cells obtained from smoke-exposed mice contained more Grx1 protein compared to cells from air-exposed controls (Figure 2b; air 18,125 RFI; CS 30,000 RFI). In line, immunohistochemistry revealed prominent Grx1 staining in alveolar macrophages of smoke-exposed mice (Figure 2c).

**Figure 3:**
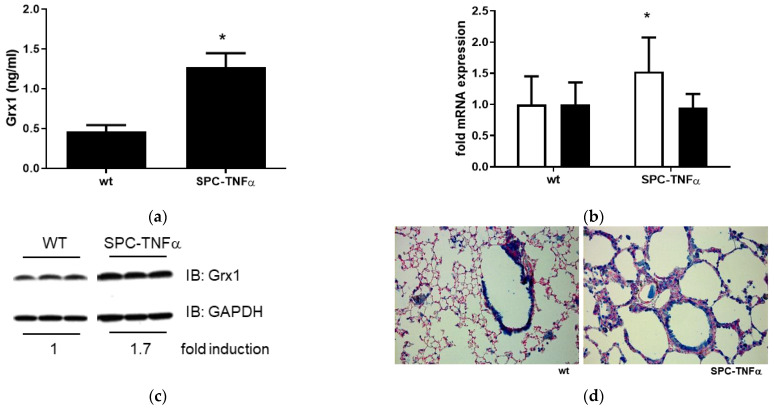
Increased expression and release of Grx1 into the BALF of SPC-TNFa mice. (**a**) BALF Grx1 levels in wt (*n* = 8) and SPC-TNFa (*n* = 11) mice. (**b**) Grx1 (white bars) and Grx2 (black bars) mRNA expression in whole lung tissue of wt (*n* = 8) and SPC-TNFa (*n* = 6) mice, corrected for RPL13A. (**c**) Grx1 protein expression in wt (*n* = 8) and SPC-TNFa (*n* = 6) mice. GAPDH was blotted as a loading control. (**d**) Representative image taken at 20× of Grx1 immunohistochemistry on lung tissue of a wt and SPC-TNFa mouse (Grx1: blue; nuclei: red). Data are expressed as mean ± SD, and * denotes *p* < 0.05 vs. representative control.

Persistent chronic inflammation in COPD patients (even after smoking cessation) is a major driver of lung function decline, as are disease exacerbations, which are hallmarked by acute inflammation. Given the observed correlations between the Grx1/PSSG axis and inflammatory cells and cytokines in sputum, it was examined whether Grx1 alterations are also present in a COPD model of chronic pulmonary inflammation. For this purpose, SPC-TNFα mice were used, which overexpress human TNFα in a SPC-dependent manner. As shown in Figure 3a, BALF from SPC-TNFα mice contained significantly higher levels of Grx1 protein compared to wild-type littermate controls. In line with the BALF data, lung tissue from SPC-TNFα mice also exhibited increased levels of Grx1 mRNA and protein compared to wild-type littermate controls (Figure 3b,c). Immunohistochemistry revealed more generalized Grx1 positivity in the alveolar region of SPC-TNFα mice, including in alveolar epithelial cells and macrophages, compared to wild-type littermates (Figure 3d).

### 3.4. Upregulation of Grx1 and Its Release by Macrophages and Epithelial Cells After Exposure to Cigarette Smoke and Inflammatory Mediators

**Figure 4:**
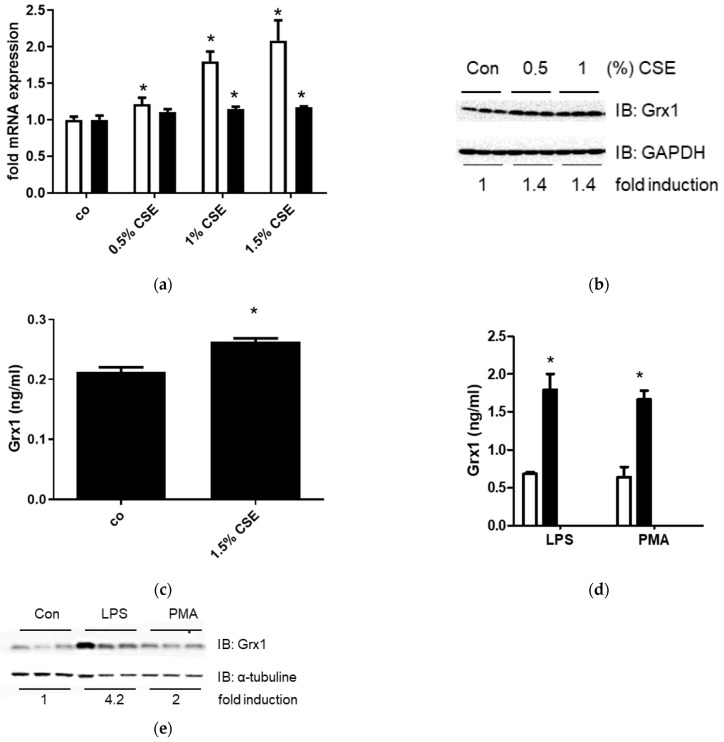
Increased expression and release of Grx1 by J774.1 cells in response to CSE, PMA and LPS. (**a**) Grx1 (white bars) and Grx2 (black bars) mRNA expression in control J774.1 cells and after 24 h of CSE exposure, corrected for RPL13A. (**b**) Grx1 protein expression in control J774.1 cells and after 24 h of CSE exposure. GAPDH was used as a loading control. (**c**) Grx1 levels in cell culture supernatant of control J774.1 cells and after 24 h of CSE stimulation. (**d**) Grx1 levels in cell culture supernatant of control J774.1 cells (white bars) and after 24 h of exposure to 10 ng/mL LPS or 5 ng/mL PMA (black bars). (**e**) Grx1 protein expression in control J774.1 cells and after 24 h of exposure to 10 ng/mL LPS or 5 ng/mL PMA. a-tubulin was used as a loading control. Data are expressed as mean ± SD, and * denotes *p* < 0.05 vs. representative control.

Our findings suggest that macrophages are a likely source of extracellular Grx1 and that its release is triggered by cigarette smoke, as well as inflammation. To further investigate this, Grx1 regulation was examined in macrophages in vitro. In line with the upregulation of Grx1 in BALF cells obtained from smoke-exposed mice, treatment of J774.1 macrophages with CSE for 24 h increased mRNA expression of Grx1 in a dose-dependent manner (Figure 4a). Minor effects on Grx2 mRNA expression were also observed. After 24 h of CSE exposure, intracellular Grx1 protein levels (Figure 4b), as well as extracellular Grx1 levels in the culture medium (Figure 4c), were significantly enhanced. Stimulation of J774.1 cells with the inflammatory mediators LPS and PMA for 24 h also enhanced Grx1 levels in the cell culture medium (Figure 4d) and upregulated intracellular Grx1 protein levels (Figure 4e). Surprisingly, treatment with TNFα did not affect Grx1 expression or release from J774.1 cells.

**Figure 5:**
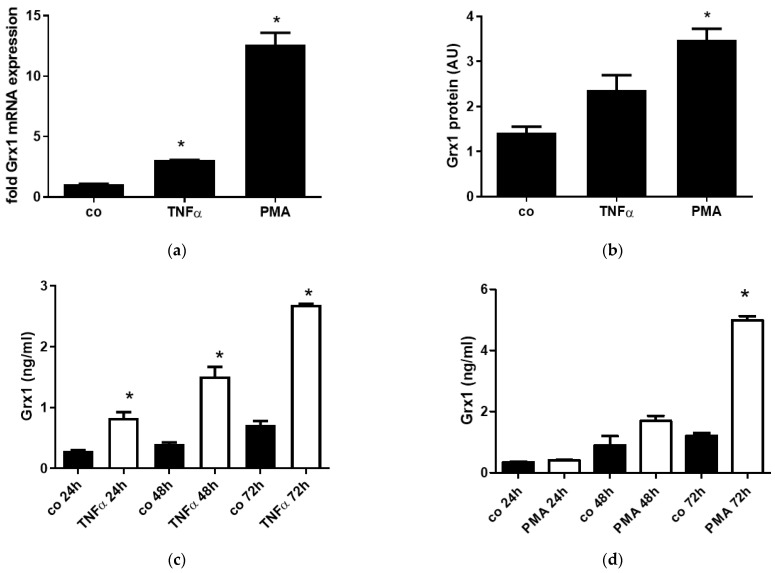
Increased expression and release of Grx1 by H292 cells in response to TNFα and PMA. (**a**) Grx1 mRNA expression in control H292 cells and after 24 h of treatment with 10 ng/mL TNFα or 5 ng/mL PMA, corrected for HPRT. (**b**) Grx1 protein expression in control H292 cells and after 24 h of treatment with 10 ng/mL TNFα or 5 ng/mL PMA. a-tubulin was used as a loading control, and data are expressed as arbitrary units. Grx1 levels in cell culture supernatant of control J774.1 cells and after 24 h of CSE stimulation. Grx1 levels in cell culture supernatant of control H292 cells and at various time points after exposure to 10 ng/mL TNFa (**c**) or 5 ng/mL PMA (**d**). Data are expressed as mean ± SD, and * denotes *p* < 0.05 vs. representative control.

Because Grx1 appeared to be upregulated in epithelial cells in the SPC-TNFα model, we examined the effects of inflammatory stimuli on Grx1 expression and release from epithelial cells in vitro. In H292 cells, enhanced Grx1 mRNA (Figure 5a) and protein (Figure 5b) expression were shown after treatment with PMA, and to a lesser extent after treatment with TNFα. Both stimuli furthermore enhanced Grx1 levels in the cell culture medium, with extracellular Grx1 accumulating progressively over a 72 h period (Figure 5c,d).

### 3.5. Uptake and Influence of Extracellular Grx1 on Macrophage MMP Expression

**Figure 6:**
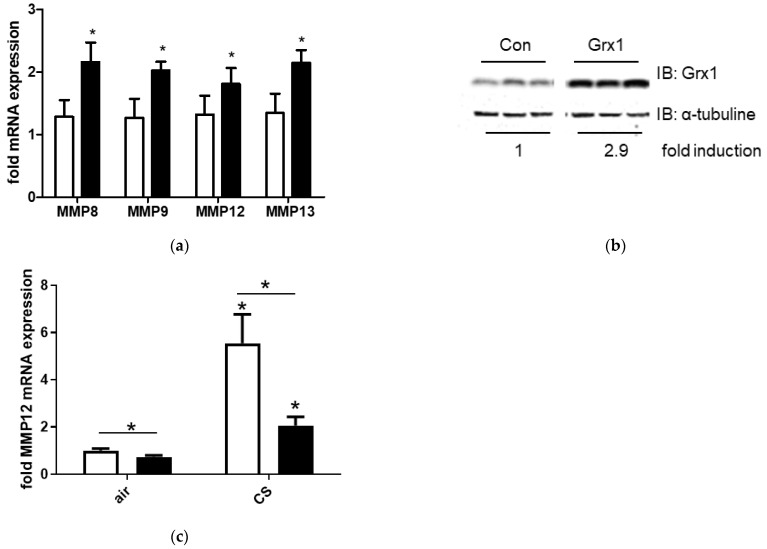
Uptake of Grx1 and influence of Grx1 on MMP expression. (**a**) mRNA expression of MMP8, MMP9, MMP12 and MMP13, relative to RPL13A in control J774.1 cells (white bars) and after 4 h of incubation with 50 ng/mL recombinant Grx1 (black bars). (**b**) Grx1 protein levels in control J774.1 cells and after 4 h of incubation with 50 ng/mL recombinant Grx1.a-tubulin was used as a loading control. (**c**) MMP12 expression in lung tissue of wt (white bars) and *Glrx1* k.o. mice (black bars) after 4 weeks of air or CS exposure, relative to RPL13A. Data are expressed as mean ± SD, and * denotes *p* < 0.05 vs. representative control.

We previously demonstrated that Grx1 exerts pro-inflammatory effects, as its overexpression enhanced NF-κB activation and the production of pro-inflammatory mediators in epithelial cells in vitro and in lung inflammation models in vivo [11,26]. Given that MMPs are regulated by NF-κB and contribute to emphysema development, we tested whether extracellular Grx1 affects MMP expression in macrophages. Treatment of J774.1 macrophages with recombinant Grx1 significantly increased the expression of MMP8, MMP9, MMP12 and MMP13 (Figure 6a). In addition, exposure to recombinant Grx1 resulted in elevated intracellular Grx1 protein levels, indicating that macrophages are able to take up intact Grx1 (Figure 6b). Lastly, *Glrx1*-deficient mice displayed attenuated basal expression of MMP12, and the absence of *Glrx1* largely prevented the increase in MMP12 expression in response to CS (Figure 6c).

## 4. Discussion

In this manuscript, we demonstrate that the Grx1-PSSG axis is markedly altered in sputum during COPD exacerbations. Sputum Grx1 levels were positively associated with smoke exposure and inflammation, and negatively associated with lung function. In contrast, PSSG levels showed opposite correlations, being negatively associated with smoking and inflammation, and positively associated with lung function. In two complementary animal models of COPD, enhanced Grx1 levels were observed in BALF, and release of Grx1 from macrophages into cell culture medium was detected after stimulation with CSE and inflammatory mediators. Lastly, Grx1 was shown to modulate MMP expression in macrophages and lung tissue.

Grx1 protein levels were previously reported to be enhanced in sputum of patients with a COPD exacerbation compared to non-smoking controls [14]. Importantly, although the sample size of the current study was relatively limited, it extends published findings by demonstrating that Grx1 is significantly increased during an exacerbation compared to the clinically more relevant stable disease state. Moreover, we identified a clear effect of smoking on sputum Grx1 levels in control subjects, and observed significant positive correlations between the number of pack years smoked and both Grx1 protein levels and Grx activity. These smoking-related effects were corroborated in a mouse model, which indicated macrophages as a likely source of extracellular Grx1. Previous immunohistochemical analysis of lung tissue of COPD patients indicated decreased numbers of Grx1-positive macrophages in very severe COPD [14]. Although extracellular Grx1 has mostly been detected from non-adherent cells by screening various cell lines [17], stimulation of macrophages with CSE in the current study enhanced Grx1 expression and release into the culture medium. Enhanced Grx1 protein levels and Grx activity were also reported in BALF of mice after oropharyngeal aspiration of LPS [26]. We now extend these findings by showing similar effects in a model of chronic lung inflammation and by demonstrating that inflammatory mediators increase Grx1 expression and secretion in macrophages and bronchial epithelial cells in vitro. Yet, the cellular sources of extracellular Grx1 in sputum remain to be determined, as Grx1 levels and Grx activity did not significantly correlate with specific cell populations in induced sputum. Previously, Grx1 was shown to be released from peripheral blood mononuclear cells, which could be enhanced by stimulation with 12-*O*-tetradecanoyl-phorbol-13-acetate (TPA) [17]. Although this is in line with macrophages being plausible contributors, their relative proportion tended to be decreased in smokers and COPD patients, whereas neutrophil counts increase during an exacerbation [27]. These observations argue against macrophages as a prominent source of extracellular Grx1. In asthma, we similarly observed no clear association between Grx1 and specific sputum cell types; however, Grx1 was specifically elevated in patients classified as eosinophilic or paucigranulocytic [18]. Moreover, primary epithelial cells isolated from asthma patients expressed higher Grx1 levels compared to cells isolated from controls, and we show here that epithelial cells release Grx1 upon inflammatory stimulation. While immunohistochemical analysis of lung tissue indicated weak positivity in bronchial epithelium [28], it did not reveal elevated Grx1 expression in epithelium in COPD patients [14]. In a pilot study, we observed similar Grx1 mRNA expression in primary bronchial epithelial cells isolated from COPD patients vs. controls, which is in agreement with the unaltered sputum Grx1 levels observed between stable COPD and controls. Epithelial cells remain a plausible source of extracellular Grx1 during periods of acute inflammation, since inflammatory mediators triggered the release of Grx1 from epithelial cells. Future studies are needed to clarify the cellular origin(s) of extracellular Grx1, as well as secretion mechanisms. Indeed, since Grx1 lacks an N-terminal signal peptide for classical secretion via the ER-Golgi pathway, secretion must involve either vesicle- or carrier-mediated mechanisms. The mechanisms and consequences of Grx1 uptake by macrophages in vitro, previously also reported for epithelial cells [29], also warrant further investigation.

The observed increase in Grx1 protein levels was paralleled by increased total Grx activity in smokers and in COPD patients during exacerbations. Importantly, in line with the function of Grx to remove GSH from proteins, PSSG levels in sputum were decreased during an exacerbation, and inversely correlated with both Grx1 protein levels and total Grx activity. Since the association of PSSG with Grx1 levels was stronger compared to the association with Grx activity, it is likely that not all extracellular Grx1 in sputum is enzymatically active, including during an exacerbation. The Grx1 immunoreactivity with a lower than expected MW seen in the majority of sputum samples obtained during an exacerbation is intriguing. We speculate that this represents a degradation product of Grx1. Although this remains to be formally tested, caspase-3 and -8 have previously been shown to cleave human Grx1 to an approximately 8 kDa fragment following Fas ligand stimulation in lung epithelial cells [30]. Of note, also in sputum of approximately 30% of asthmatics, a similar lower MW form of Grx1 was detected [18].

PSSG can be considered a reservoir of GSH, which can be liberated by Grx1 to increase the antioxidant potential of cells and tissues. The combination of decreased PSSG levels and enhanced Grx1 levels and Grx activity in sputum during an exacerbation may represent an adaptive response to elevated oxidative stress, aimed at mobilizing GSH from proteins. This is furthermore supported by the significant negative associations between PSSG and Grx1 and Grx activity. Unfortunately, GSH and GSSG levels were not assessed in the current study to support this hypothesis. The positive correlations between sputum PSSG levels and macrophages and epithelial cells suggest that these cells may contribute to extracellular PSSG and/or GSH. Epithelial cells are indeed a known prominent source of extracellular GSH [31]. Importantly, PSSG also protects protein thiols from further oxidation that is irreversible. This protective function is supported by the positive correlations between sputum PSSG levels and lung function parameters. Moreover, the decreased levels of PSSG as seen during an exacerbation could negatively affect the proteins involved, leading to irreversible oxidation and damage, and to their accumulation or degradation [32,33,34]. The negative association between PSSG levels and neutrophil percentages, as well as pack years smoked, might indeed reflect oxidative protein damage elicited by neutrophils and smoke [35,36]. Thirdly, the function of various proteins, especially those with conserved and functionally important cysteine residues, can be modified by PSSG. Grx1 modulates the function of proteins by catalyzing protein deglutathionylation and thereby exerts redox control over cellular signalling. As such, Grx1 has previously been shown to modulate pulmonary inflammation. *Glrx1*-deficient mice exhibit reduced LPS-induced NF-κB activation and inflammation [26], and are protected from smoke-induced neutrophilia [16]. In a house dust mite model of allergic airway disease, on the other hand, *Glrx1* deficiency resulted in enhanced neutrophilic and Th-17 responses, but attenuated eosinophilia and Th-2 responses, accelerating resolution of airways hyperresponsiveness and mucus metaplasia [37]. These findings underscore the context-dependent role of Grx1 in pulmonary inflammation. In our study, sputum Grx1 protein levels negatively correlated with lung function parameters. It could be speculated that Grx1 released into the extracellular milieu may promote disease progression and lung function deterioration associated with exacerbations. These disease-promoting effects are likely elicited through the modulation of PSSG, as PSSG levels in sputum were positively associated with lung function parameters and inversely with Grx1 levels. While most studies to date have focused on intracellular proteins regulated by PSSG [9], several extracellular proteins, such as S100A9 and IL-1β, are also modulated by PSSG [38,39]. The observed negative associations between PSSG and IL-1β and IL-8 are consistent with anti-inflammatory effects of PSSG. Also within macrophages, Grx1 and PSSG modulate a range of processes important for their functionality, including differentiation and polarization, cellular metabolism, production of inflammatory mediators [40,41,42], and, as we demonstrate here, expression of MMPs. Although our findings demonstrate that this redox axis is potentially linked to tissue remodelling and emphysema development, they should be corroborated in larger studies, which should take into account different COPD phenotypes and patient heterogeneity.

## 5. Conclusions

In conclusion, this study highlights the potential importance of the Grx1–PSSG redox axis in COPD, especially during acute exacerbations. Future research should examine in more detail the intricate relation between lung function and inflammation.

## 6. Patents

N.L.R. holds patents regarding The Detection of Glutathionylated Proteins (US Pat.Apl.Ser.No 11/698,300) and Treatments Involving Glutaredoxins and Similar Agents (US Pat.Apl.Ser.No 60/934,129).

## Figures and Tables

**Table 1:** Subject characteristics.

	Control Non-Smokers	Control Smokers	Stable COPD	COPD Exacerbation
Number of subjects	12	12	16	14
Age	49.8 ± 10.3	53.9 ± 10.2	58.6 ± 9.6	63.9 ± 8.7 ^#^
Sex, M/F (*n*)	6/6	7/5	12/4	12/2
Pack years	0	19.9 ± 18.3 ^#^	44.5 ± 11.1 *^,#^	41.5 ± 18.4 *^,#^
BMI	26 ± 5.1	24.8 ± 3.1	26.3 ± 4.5	27.3 ± 5.6
FEV_1_% predicted	101.4 ± 17.6	95.5 ± 17.8	49.2 ± 14.5 *^,#^	43.2 ± 17.7 *^,#^
FVC % predicted	105.4 ± 13.8	107.7 ± 21.2	82.6 ± 22.3 *^,#^	69.4 ± 22.3 *^,#^
FEV_1_/FVC	80.0 ± 6.2	75.6 ± 9.6	49.9 ± 9.5 *^,#^	47.2 ± 9.5 *^,#^
GOLD II, III, IV (*n*)	-	-	10/4/2	6/5/3
% sputum macrophages	60 (13–96)	26 (2–75)	11 (0–77)	8 (0–32)
% sputum neutrophils	18 (0–84)	58 (8–94)	82 (3–95) *	84 (26–100)
% sputum eosinophils	0 (0–3.2)	0 (0–5.8)	18 (0–5)	0.8 (0–23.3)
% sputum lymphocytes	1.8 (0–3.4)	1.6 (0–10)	0.5 (0–3)	1.6 (0–6.3)
Sputum IL-1b (pg/mL)	1.2 (0.43–8.6)	1.5 (0.4–3.8)	1.3 (0.4–31.2)	3.2 (0.6–602.6) ^$^
Sputum IL-8 (pg/mL)	26 (7–132)	79 (16–244)	187 (12–3989)	333 (60–1990)

Data are expressed as mean ± SD or median (IQR), * represents *p* < 0.05 vs. control smokers, ^#^ represents *p* < 0.05 vs. control non-smokers, ^$^ represents *p* < 0.05 vs. stable COPD. Pack years was not available from 1 stable COPD patient and 8 control smokers.

## Data Availability

The original contributions presented in this study are included in the article/Supplementary Materials. Further inquiries can be directed to the corresponding author.

## Supplementary Materials

The following Supporting Information can be downloaded at: https://www.mdpi.com/article/10.3390/antiox15030330/s1, Figure S1: Correlations between PSSG and Grx1 protein and activity levels; Table S1: Primer sequences.

## Abbreviations

The following abbreviations are used in this manuscript:

BAL(F)	Broncho alveolar lavage (fluid)
COPD	Chronic obstructive pulmonary disease
CS(E)	Cigarette smoke (extract)
Grx	Glutaredoxin
PSSG	Protein S-glutathionylation
RT	Room temperature
