# Supplementary material for: Sputum Glutaredoxin 1 and Protein S-Glutathionylation in COPD
Source: Antioxidants (Basel). 2026 Mar 6;15(3):330. doi: 10.3390/antiox15030330 (PMC13023451; doi:10.3390/antiox15030330)
Supplement: Supplementary file 1 [file antioxidants-15-00330-s001.zip › antioxidants-4141195-supplementary.pdf]

Table S1: Primer sequences.

	Forward primer	Reverse primer
Mouse targets		
Glrx1	TTTACAACAGCTCACCGGAG	TCACTGCATCCGCCTATG
Glrx2	AAATCTTCTTGGCCATGGAA	AACAGCACATCGTCGTTTTG
MMP8	CCCAGTACCTGAACACCTGGAA	TTAAGCTTCTCTGCAACCATCGT
MMP9	TCTTCCCCAAAGACCTGAAAAC	GCCCGGGTGTAACCATAGC
MMP12	TGAGGCAGAAACGTGGACTAAA	ATTGACTTTGGATTATTGGAATGCT
MMP13	AATCTATGATGGCACTGCTGACA	GTTTGGTCCAGGAGGAAAAGC
RPL13A	CACTCTGGAGGAGAAACGGAAGG	GCAGGCATGAGGCAAACAGTC
HPRT	TGGATATGCCCTTGACTATAATGAGTAC	AGGACTCCTCGTATTTGCAGATTC
Human targets		
Glrx1	TCAGTCAATTGCCCATCAAA	AGATCACTGCATCCGCCTAT
Glrx2	TTTACAAATGACTGGTGAAAGAAC	TGTCAGTTGCACCTCCAATAA
HPRT	AGAATGTCTTGATTGTGGAAGA	ACCTTGACCATCTTTGGATTA

Supplemental figures

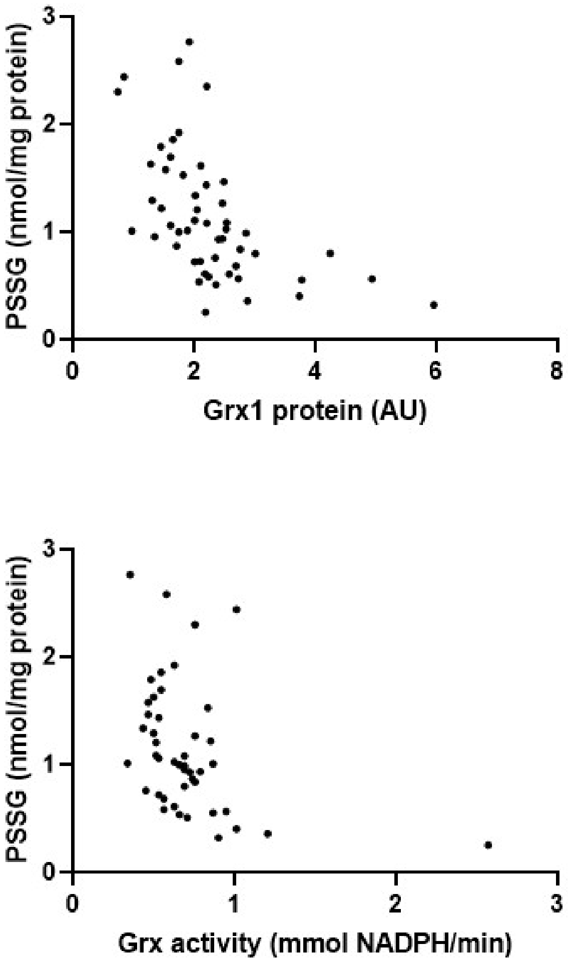

Figure S1. Correlations between PSSG and Grx1 protein and activity levels.
